# The Forgotten Lumbocostal Ligament: Anatomical Study with Application to Thoracolumbar Surgery

**DOI:** 10.7759/cureus.925

**Published:** 2016-12-11

**Authors:** Erfanul Saker, Gabrielle G Tardieu, Fernando Alonso, Beom Sun Chung, Christian Fisahn, Marios Loukas, Rod J Oskouian, R. Shane Tubbs

**Affiliations:** 1 Department of Anatomical Sciences, St. George's University School of Medicine, Grenada, West Indies; 2 Neurosurgery, University Hospitals of Cleveland, Case Medical Center; 3 Neurosurgery, Swedish Neuroscience; 4 Department of Anatomy, Ajou University School of Medicine; 5 Orthopedic Surgery, Swedish Neuroscience Institute; 6 Department of Trauma Surgery, BG University Hospital Bergmannsheil, Bochum, Germany; 7 Neurosurgery, Complex Spine, Swedish Neuroscience Institute; 8 Neurosurgery, Seattle Science Foundation

**Keywords:** anatomy, spine, surgery, ligamentous

## Abstract

Introduction: Most ligaments of the human body have been well studied. However, the lumbocostal ligament has received little attention in the extant medical literature and, to our knowledge, has not undergone anatomical study. Therefore, the present study was performed to better characterize this structure’s anatomy and relationships.

Methods: In the prone position, 10 adult cadavers underwent dissection of their lumbocostal ligaments. All specimens were unembalmed and had no history of surgery to the spine. The lumbocostal ligament was dissected and measurements made using calipers and a ruler. This ligament’s attachments were determined as well as its relationships to surrounding fasciae, muscle, and nerves.

Results: A lumbocostal ligament was identified on all sides. The ligament was posterior to the quadratus lumborum muscle on all sides. The mean length of the ligament was 3 cm. The overall shape of the ligaments ranged from short bands to large rhomboidal sheets. Inferiorly, the lumbocostal ligament blended with the middle layer of the thoracolumbar fascia on all sides. The ligament attached to the transverse processes of L1 on 25% of sides and onto the transverse processes of L1 and L2 on 75% of sides. The ligament became taut with rib elevation and was lax with rib depression.

Conclusions: The lumbocostal ligament is a constant structure of the thoracolumbar junction. Appreciation of this ligament can help localize the transverse processes of L1 and L2 and adjacent nerves, such as the regional dorsal rami as they exit near its attachment onto the lumbar transverse processes.

## Introduction

**Figure 1:**
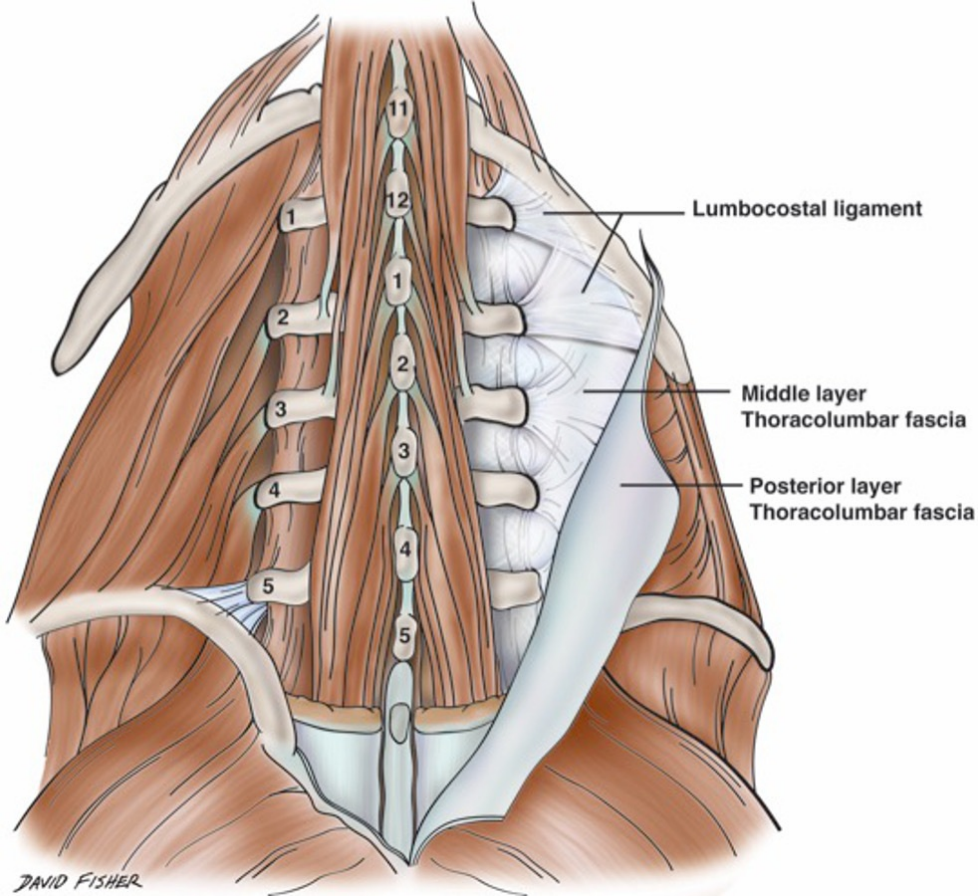
Schematic view of the posterior lower back Note the lumbocostal ligament and its relationships to regional bony anatomy and fascial layers. Figure courtesy of Hand-Atlas Der Anatomie Des Menschen, 1864.

The lumbocostal ligament (vertebrocostal ligament or ligament of Henle) has been underrepresented in the medical literature and joins the twelfth rib to the lumbar spine (Figure 1). Moreover, descriptions of this ligament have varied. For example, some sources state that this fibrous band arises from the transverse process of the first lumbar vertebra and others state that it arises from the transverse processes of the first and second lumbar vertebrae [1-2]. The formation of the ligament begins from the costal processes of the lumbar vertebrae. Here, reinforced fiber strands radiate in a fan-shaped configuration into the middle layer of the thoracolumbar fascia, forming at the upper end the lumbocostal ligament [3]. Along the same path as the ligament is the quadratus lumborum muscle, which lies lateral and posterior to the psoas major muscle [4]. This flat muscle inserts onto the lower border of the twelfth rib, fixing it in place to assist the action of the diaphragm in inspiration.  

To our knowledge, no previous anatomical studies exist on the lumbocostal ligament. With such a lack of information on this structure in the literature and controversial descriptions, the current anatomical study was performed.

## Materials and methods

In the prone position, 10 adult cadavers (20 sides, six males/four females) aged 58 - 89 years at death (mean: 77 years) underwent dissection of their lumbocostal ligaments. All specimens were unembalmed and had no history of surgery to the spine. A skin incision was made from the sacrum to the mid-thorax. Next, the posterior layer of the thoracolumbar fascia was identified and cut from the midline spinous processes. This layer and associated muscles (e.g., latissimus dorsi) were reflected laterally. The underlying erector spinae muscle was then identified, cut, and reflected inferiorly. The twelfth rib and middle layer of the thoracolumbar fascia were identified. The lumbocostal ligament was dissected and measurements made using calipers and a ruler. This ligament’s attachments were determined as well as its relationships to surrounding fasciae, muscle, and nerves. Statistical analyses between sides and sex were performed with Statistica™ for Windows (Dell, Inc., Tulsa, OK) with significance set at p < 0.05.

No informed patient consent was necessary for this cadaver study.

## Results

**Figure 2:**
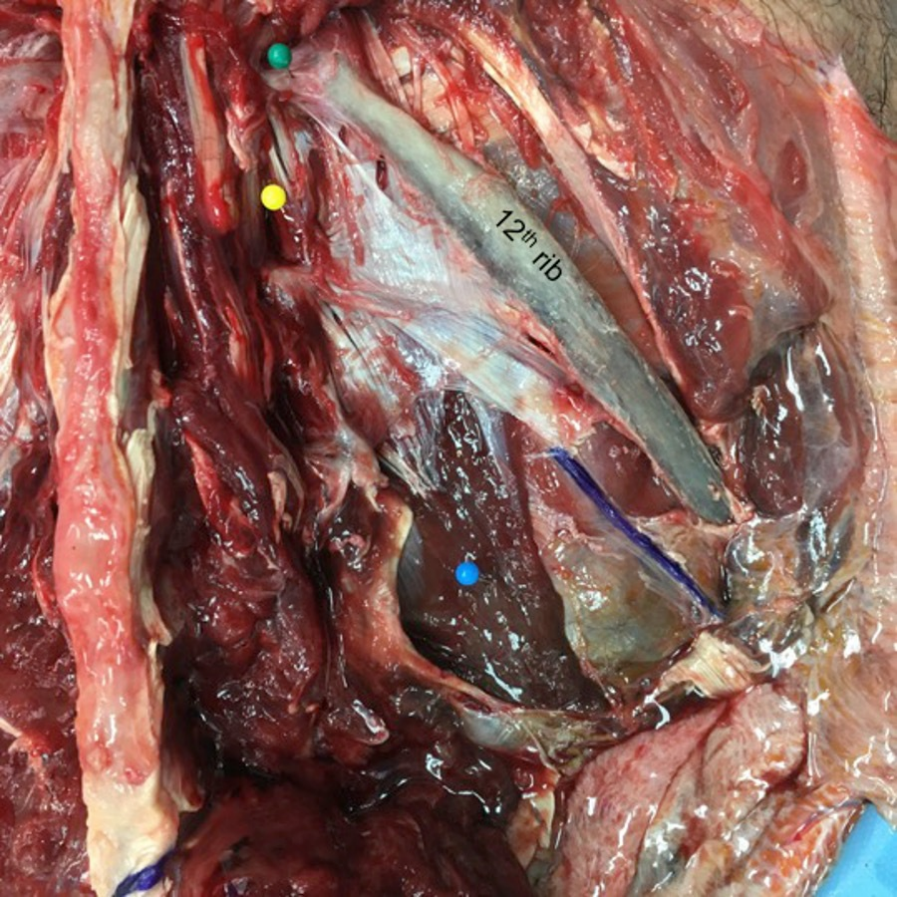
Dissection of the right lumbocostal ligament Note its attachment along the lower margin of the 12th rib and relationships to the intertransversarii muscle (yellow pin) and quadratus lumborum muscle (blue pin). Also, note the exit site of the subcostal nerve colored purple.

**Figure 3:**
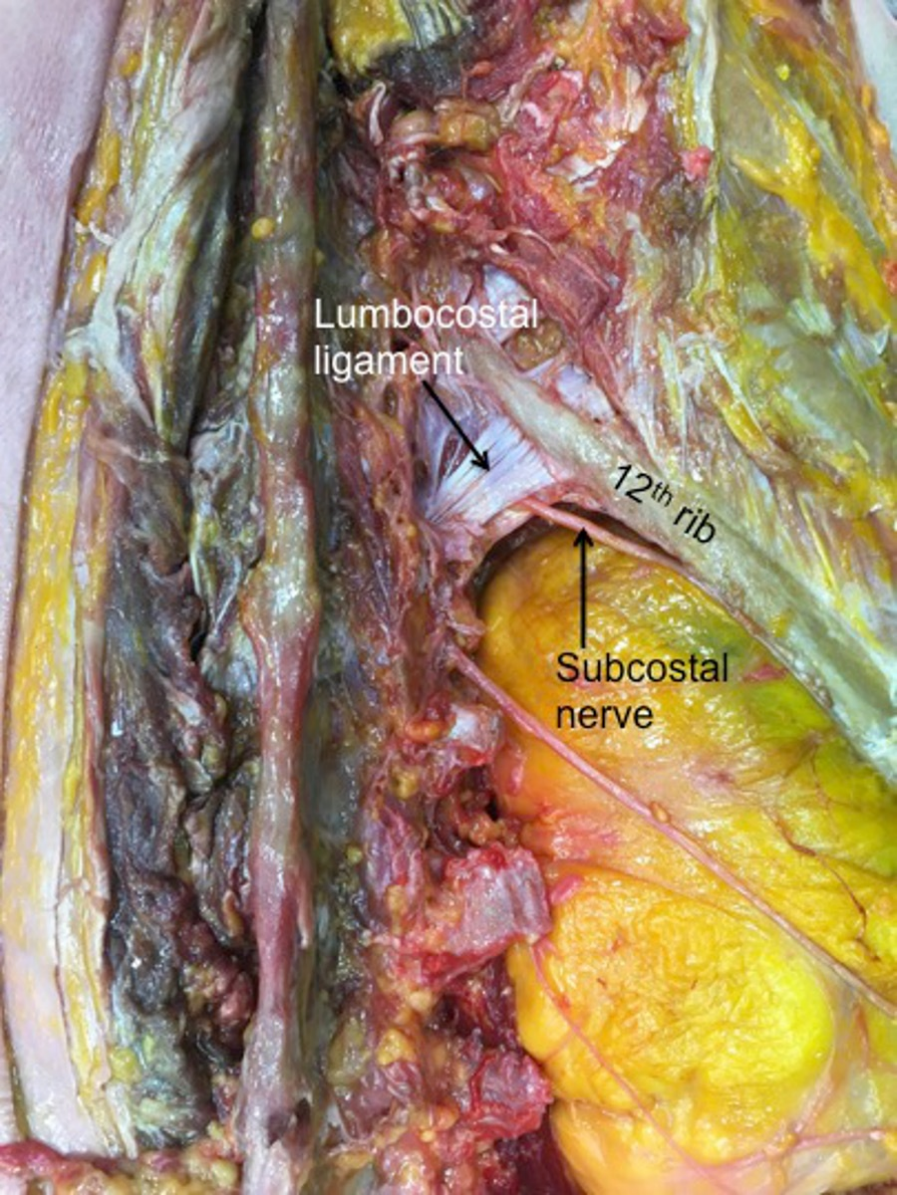
Right lumbocostal ligament Compare this smaller version of the ligament to the broader morphology seen in Figure 2. On this specimen, the ligament attaches to the L1 transverse process. Also, note the intimate relationship between the ligament in this specimen and the subcostal nerve.

**Figure 4:**
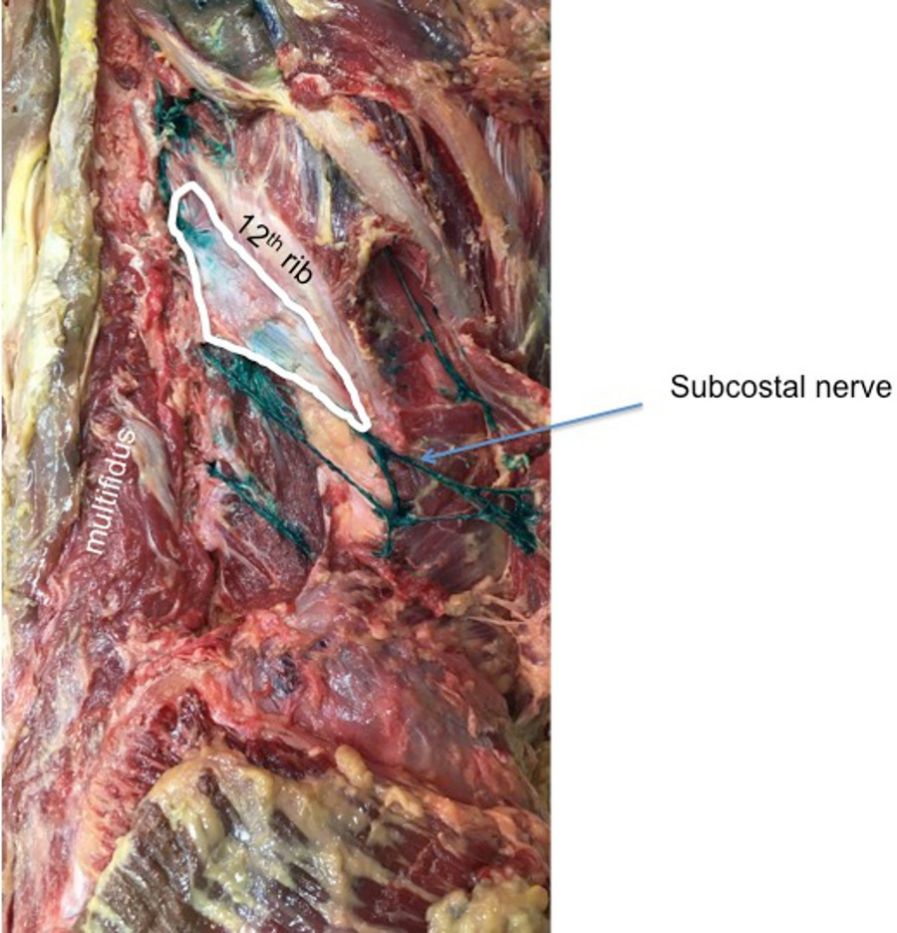
Right lumbocostal ligament with a triangular shape For reference, note the lumbar multifidus muscle and relationship between the ligament and the subcostal nerve colored green on this specimen.

**Figure 5:**
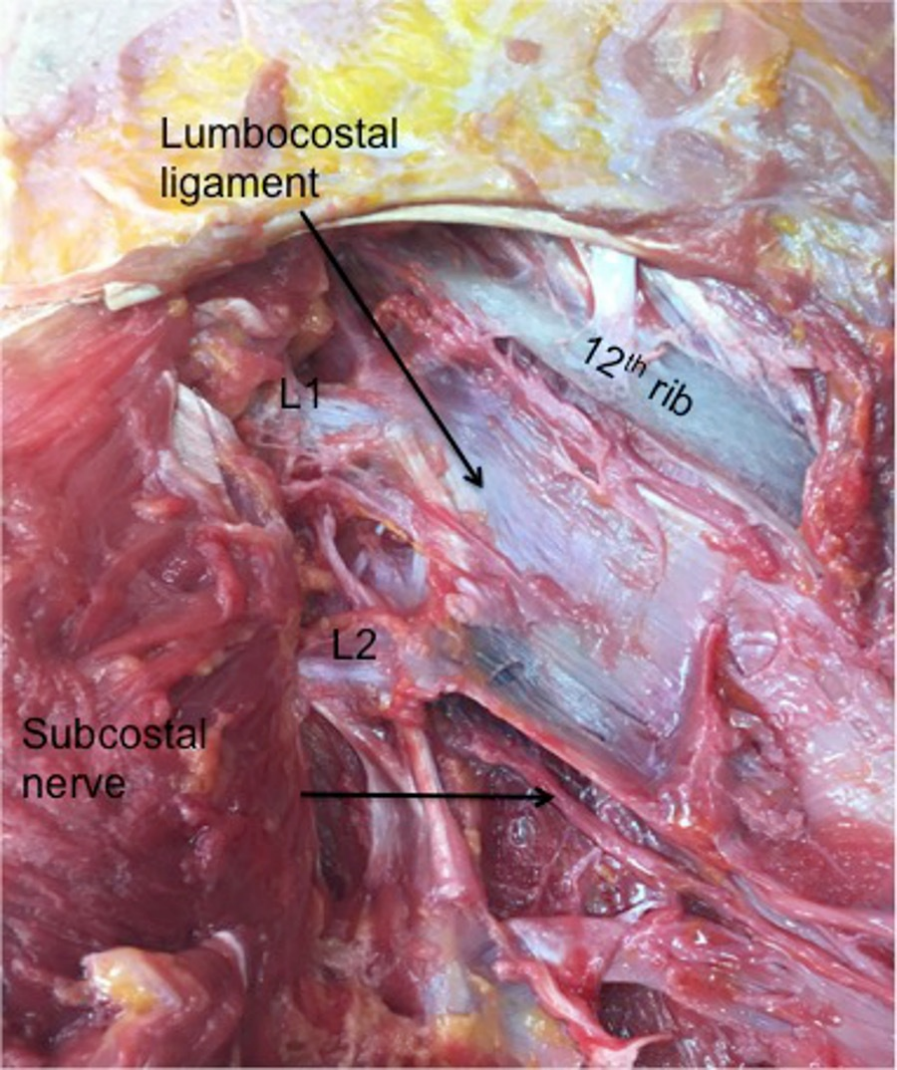
Right lumbocostal ligament Note the attachments in this specimen onto both the L1 and L2 transverse processes.

**Figure 6:**
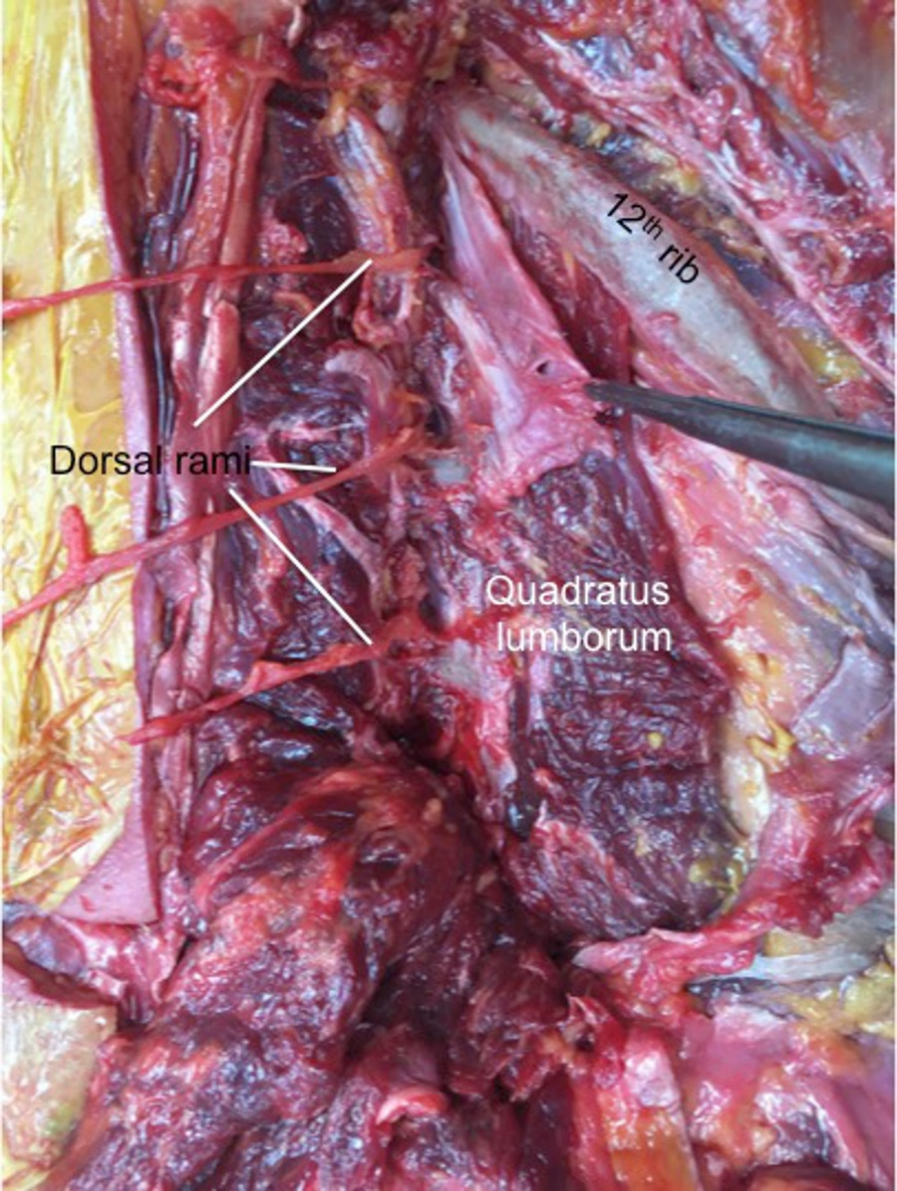
Right lumbocostal ligament (forceps) detached from the underside of the 12th rib to show its relationship to the underlying quadratus lumborum muscle Note the exiting dorsal rami near the L1 and L2 attachments of the ligament.

A lumbocostal ligament was identified on all sides. The ligament was posterior to the quadratus lumborum muscle on all sides and the intertransversarii muscles were posterior to its transverse process attachment (Figure 2). The length of the ligaments was variable (range: 1 – 4.5 cm; mean: 3 cm). The fiber direction was also varied with smaller ligaments tending to run in a medial to lateral direction (Figure 3) and larger ligaments having a more transverse orientation (Figure 4). The overall shape of the ligaments ranged from short bands (Figure 3) to large rhomboidal sheets (Figures 2, 5). Some ligaments were more triangular in shape (Figure 4). Inferiorly, the lumbocostal ligament blended with the middle layer of the thoracolumbar fascia on all sides. In fact, the lumbocostal ligament, based on our dissections, could be considered a thickened superior part of the middle layer of the thoracolumbar fascia as it attaches to the transverse processes of L1 (five sides, 25%) or L1 and L2 (15 sides, 75%) transverse processes. On two sides (10%), a small branch of the subcostal nerve was found to pierce the lumbocostal ligament. The ligament became taut with rib elevation and was lax with rib depression. The subcostal nerve was deep to the lumbocostal ligament on nine sides (45%) (Figure 3) or traveled along its lower border (11 sides, 55%) (Figures 2, 4-5). The lumbocostal ligament was a good landmark for the subcostal nerve and adjacent dorsal rami as they exit near its attachment onto the lumbar transverse processes (Figure 6). No statistical differences were noted for size or attachments between sides or sex. 

## Discussion

We found a variety of shapes for the lumbocostal ligament and that not all ligaments attached just to the L1 transverse process as stated by many authors [5]. Our study also identified a previously unreported branch of the subcostal nerve that pierced the ligament although the destination of this branch could not be ascertained.

### Clinical relevance 

The lumbocostal ligament is near the superior lumbar triangle (Grynfeltt’s triangle) [3, 6]. This region, believed to arise when the serratus posterior inferior is poorly developed [3], is supported by the thoracolumbar fascia from the internal oblique muscle and the transversus abdominis and is covered by the latissimus dorsi [6]. The triangle is bound laterally by the posterior border of the internal abdominal oblique, medially by the quadratus lumborum, and superiorly by the twelfth rib and the lumbocostal ligament [6]. The superior lumbar triangle represents the peritoneal opening of the musculoaponeurotic tunnel and constitutes a weak area in the posterior abdominal wall, making it a potential space for herniation [3, 6]. Theoretically, larger lumbocostal ligaments might decrease the chances of herniation in this area, although future studies would need to demonstrate this.        

### Surgery

Approaches to the retroperitoneal space often use an oblique lumbar incision that begins at the lateral margin of the erector spinae muscle located at the level of the twelfth rib. Next, the latissimus dorsi and the external oblique aponeurosis are divided. As the surgeon proceeds layer by layer, the lumbocostal ligament will also be divided [7]. This ligament can also be used as a landmark in the muscle-sparing lumbar incision approach to the kidney [7]. Additionally, the lumbocostal ligament is involved in the dissection of the flap from the transversus abdominis and thoracolumbar fascia when a nephropexy technique is utilized to cover the surface of the kidney [8]. With a better understanding of this ligament, its size and relationships to the subcostal nerve and adjacent dorsal rami might be seen on preoperative MRI and help to better tailor various approaches to this region.

## Conclusions

The lumbocostal ligament is a constant structure of the thoracolumbar junction. Appreciation of this ligament can help localize the transverse processes of L1 and L2 and adjacent nerves, such as regional dorsal rami as they exit near its attachment onto the lumbar transverse processes. Further biomechanical studies are now necessary to evaluate its true function, tensile strength, and potential role in such pathologies as twelfth rib syndrome. 
